# Simvastatin reduces atherogenesis and promotes the expression of hepatic genes associated with reverse cholesterol transport in apoE-knockout mice fed high-fat diet

**DOI:** 10.1186/1476-511X-10-8

**Published:** 2011-01-18

**Authors:** Guohua Song, Jia Liu, Zhenmei Zhao, Yang Yu, Hua Tian, Shutong Yao, Guoli Li, Shucun Qin

**Affiliations:** 1 Institute of Atherosclerosis, Taishan Medical University, Taian, Shandong 271000, China; 2 Medical College, Yangzhou University, Yangzhou, Jiangsu 225009, China

## Abstract

**Background:**

Statins are first-line pharmacotherapeutic agents for hypercholesterolemia treatment in humans. However the effects of statins on atherosclerosis in mouse models are very paradoxical. In this work, we wanted to evaluate the effects of simvastatin on serum cholesterol, atherogenesis, and the expression of several factors playing important roles in reverse cholesterol transport (RCT) in apoE-/- mice fed a high-fat diet.

**Results:**

The atherosclerotic lesion formation displayed by oil red O staining positive area was reduced significantly by 35% or 47% in either aortic root section or aortic arch en face in simvastatin administrated apoE-/- mice compared to the control. Plasma analysis by enzymatic method or ELISA showed that high-density lipoprotein-cholesterol (HDL-C) and apolipoprotein A-I (apoA-I) contents were remarkably increased by treatment with simvastatin. And plasma lecithin-cholesterol acyltransferase (LCAT) activity was markedly increased by simvastatin treatment. Real-time PCR detection disclosed that the expression of several transporters involved in reverse cholesterol transport, including macrophage scavenger receptor class B type I, hepatic ATP-binding cassette (ABC) transporters ABCG5, and ABCB4 were induced by simvastatin treatment, the expression of hepatic ABCA1 and apoA-I, which play roles in the maturation of HDL-C, were also elevated in simvastatin treated groups.

**Conclusions:**

We demonstrated the anti-atherogenesis effects of simvastatin in apoE-/- mice fed a high-fat diet. We confirmed here for the first time simvastatin increased the expression of hepatic ABCB4 and ABCG5, which involved in secretion of cholesterol and bile acids into the bile, besides upregulated ABCA1 and apoA-I. The elevated HDL-C level, increased LCAT activity and the stimulation of several transporters involved in RCT may all contribute to the anti-atherosclerotic effect of simvastatin.

## Background

Statins are class of drug used to lower cholesterol levels by inhibiting the enzyme 3-hydroxy-3-methyl-glutaryl-CoA reductase (HMGCR), which plays a central role in the production of cholesterol in the liver. Increased cholesterol levels have been associated with cardiovascular diseases (CVD), and statins are therefore first-line pharmacotherapeutic agents for the treatment of these diseases in humans. Recently a great line of animal species have been used to study the pathogenesis and potential treatment of the lesions of atherosclerosis. However until now the effects of statins in animal models of atherosclerosis are not very consistent. The apoE-knockout (apoE-/-) mouse is a well-established genetic mouse model of atherogenic hypercholesterolemia, in which mice spontaneously develop atherosclerosis with similar features to those observed in human type III familial hyperlipoproteinemia. Florian Bea et al. has reported simvastatin increased serum cholesterol and atherosclerotic lesion size in apoE-/- mice fed a chow diet although the plaque stability was increased [1]. In this work, we wanted to evaluate the effects of simvastatin on serum cholesterol and atherogenesis in apoE-/- mice fed a high-fat diet.

Reverse cholesterol transport (RCT) is believed to be crucial for preventing atherogenesis and hence the development of most cardiovascular diseases. This anti-atherogenic mechanism involves export of cholesterol from lipid-laden macrophages in the artery wall back to the liver for eventual excretion. The process of RCT can be divided into three stages: 1) the efflux of cellular cholesterol to high-density lipoprotein (HDL) from peripheral cells. Macrophage ATP-binding cassette transporter (ABC) A1, ABCG1 and scavenger receptor class B type I (SR-BI) all play important roles in the first stage. 2) the transport of HDL-cholesterol (HDL-C) in blood to the liver. The cholesterol in HDL is converted to cholesteryl esters (CE) by the enzyme lecithin-cholesterol acyltransferase (LCAT) and carried as CE in the core of the HDL particle to the liver. 3) the delivery of cholesterol esters to hepatocytes from HDL [2]. After uptaking of HDL-accociated cholesterol into hepatocytes by heptic SR-BI, secretion of cholesterol, bile acids, and phospholipid into the bile is regulated by the respective transporters ABCG5, ABCG8, ABCB4 and ABCB11. Simvastatin has been reported to improve RCT in type 2 diabetic patients with hyperlipidemia [3]. However, the effects of simvastatin on the important factors playing roles in RCT has not been elucidated. In the present study, we hypothesized that simvastatin might promote the expression of several factors involved in RCT. Therefore, we investigated the effects of simvastatin on the expression of several transporters, including macrophage ABCA1, ABCG1 and SR-BI, hepatic SR-BI, ABCG5, ABCG8, ABCB4 and ABCB11. Besides transporters, we also detected the effects of simvastatin on plasma LCAT activities and apoA-I concentrations, which also play roles in RCT in apoE-/- mice.

## Methods

### Animals and Experimental Design

21 male apoE-/- mice were purchased from laboratory animal center of Academy of Military Medical Sciences (Beijing). All experiments were approved by the laboratory animals' ethical committee of Taishan Medical University and followed national guidelines for the care and use of animals. At 5 weeks of age, the 21 apoE-/- mice were randomly divided into 2 groups, the model group (*n *= 11, vehicle treated group) and the simvastatin (5 mg/kg/d) treated group (*n *= 10). All groups were fed an atherogenic high-fat diet (15.8% fat and 1.25% cholesterol) supplemented with vehicle or simvastatin for 6 weeks. Vehicle or simvastatin were administered intragastrically once daily.

### Plasma analyses

After 6 weeks of treatment, blood was collected from the retro-orbital sinus of the mice without dietary exposure for 12 h. Concentrations of plasma total cholesterol (TC), HDL-C, and triglycerides (TG) were determined by enzymatic methods (BioSino). Non-HDL-C was calculated as TC minus HDL-C. Plasma concentrations of apolipoprotein A-I (apoA-I) was determined by ELISA kit (BlueGene). Endogenous lecithin-cholesterol acyltransferase (LCAT) activity was measured as the utilization rate of free cholesterol (FC) in native plasma according to the method by Ly et al. [4]. The FC content was measured colorimetrically by using a kit (Biovision) in pentuplicate by a microplate reader (Tecan) at zero time and after 1 hr at 37°C. LCAT activity was expressed as nanomoles FC consumed per hr per ml plasma.

### Lesion Analysis

The proximal aorta attached to the heart was used to prepare cross-sections. Cryosections (8 μm) were cut from the site where the aorta valve cups appear at the aorta root, collected and stained with oil red O and brilliant green. The volume of stained lipids (μm^2^) was calculated from five sections for an animal. For determination of atherosclerotic plaque area in aortic arch, the aortic arch were dissected and stained en face with oil red O. Quantitative analysis of plaque area was performed by 2 blinded observers using Image-Pro Plus software 4.5 (Media Cybernetics).

### Western Blots

Tissues or cells were harvested and protein extracts prepared as previously described [5]. They were then subjected to western blot analyses using anti-ABCA1, ABCG1, SR-BI, apoA-I, and β-actin antibodies (Santa Cruz). The proteins were visualized and quantified using a chemiluminescence method (Pierce) and Quantity One (Bio-Rad) software program.

### Isolation of Peritoneal Macrophages

Three days after intraperitoneal thioglycollate injection, peritoneal macrophages were harvested from six weeks simvastatin or vehicle treated apoE-/- mice. Following collection, peritoneal macrophages were immediately frozen at -80°C until RNA or protein extraction.

### Quantitative Real-Time PCR

Total cellular RNA was isolated by TRIZOL Reagent (Invitrogen). cDNA synthesis was performed using MuLV Reverse Transcriptase (Applied Biosystems). Real-time PCR was performed using a SYBR-green PCR master mix kit (TianGen Biotech). The primers used for real-time PCR were listed in Table 1. The data was analyzed by using Rotor-gene Q software ver. 1.7 (Qiagen). Relative mRNA levels were calculated by the method of 2^-DDCt^.

**Table 1:** Primers used for real-time PCR analysis

Gene	Primer	Sequence (5`-3`)
**ABCA1**	Sense	CGTTTCCGGGAAGTGTCCTA
	Anti-sense	GCTAGAGATGACAAGGAGGATGGA
**ABCG1**	Sense	GGGAAGTTGATAAAGGATGT
	Anti-sense	GATTCGGGCTATGTATGG
**SR-BI**	Sense	ATCTGGTGGACAAATGGAA
	Anti-sense	GAAGCGATACGTGGGAAT
**apoA-I**	Sense	GGCACGTATGGCAGCAAGAT
	Anti-sense	CCCAGAAGTCCCGAGTCAAT
**apoA-II**	Sense	CCAAGGCATACTTTGAGAAGACAC
	Anti-sense	GGAGAAAACAGGCAGAAGGTAGG
**LCAT**	Sense	CCCACCAGCAGGATGAATACTAC
	Anti-sense	AGGCTATGCCCAATGAGGAA
**ABCG5**	Sense	AGCGTCAGCAACCGTGTC
	Anti-sense	AGCAGCGTGGTCTTCCCT
**ABCG8**	Sense	TTAAGCCACTCCCAATACA
	Anti-sense	GTTGCTCCAAGAATAAATGA
**ABCB11**	Sense	CAAATAAGGTTGTGGGTAA
	Anti-sense	AGGACTGACAGCGAGAAT
**ABCB4**	Sense	CCCCACAGAGGGTAAGAT
	Anti-sense	CCAACCAGGGTGTCAAAT
**LXRα**	Sense	TTTGAGCAGCGTCCATTC
	Anti-sense	GCAGTCAGTGAGCCTTCG
**GAPDH**	Sense	TGACGTGCCGCCTGGAGAAA
	Anti-sense	AGTGTAGCCCAAGATGCCCTTCAG

### Statistical analysis

Statistical analysis was performed by one-way analysis of variance (ANOVA) test with the GraphPad Prism programme ver.4.0. Results are expressed as means ± SD. P values less than 0.05 were considered significant.

## Results

### Simvastatin decreases atherosclerotic lesion formation in apoE-/- mice fed a high-fat diet

**Figure 1:**
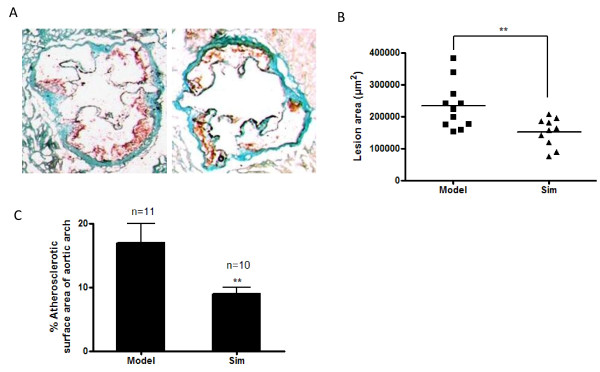
**Simvastatin inhibits atherosclerotic lesion formation in apoE-/- mice fed a high-fat diet**. A, Representative of Oil Red O-stained aortic sections (10× magnification). B, Quantitation of lesion areas in Oil Red O-stained aortic sections by Image-Pro Plus software. C, Quantification of atherosclerotic lesions in the en-face of the aortic arch. ** P < 0.01 versus model group.

As shown in Figure 1, administration of simvastatin with 5.0 mg/kg significantly inhibited the formation of atherosclerotic lesions in cross-sections of the aortic valve area by 35% compared with model group (Figure A and B). En-face oil-red O staining of the aortic arch also revealed a significant 47% reduction of atherosclerotic plaque formation in simvastatin-treated animals (Figure C). The inhibitory effect of simvastatin with doses less than 5.0 mg/kg on the formation of atherosclerotic lesions was not so obvious as that of 5.0 mg/kg of the medication (data not shown).

### Effect of simvastatin on plasma lipoprotein profiles and plasma LCAT activities

**Figure 2:**
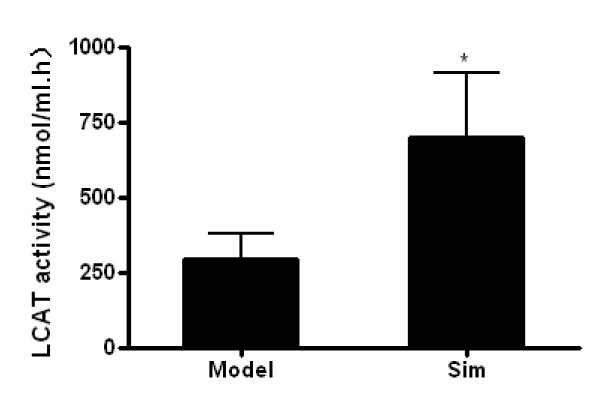
**Effect of simvastatin on endogenous LCAT activity in plasma**. LCAT activity was measured as the utilization rate of FC in native plasma. LCAT activity was expressed as nanomoles FC consumed per hr per ml plasma. Data are expressed as means ± SD (n = 10). * P < 0.05 versus model group.

As shown in Table 2, plasma TC, TG, and non-HDL-C levels after 6 weeks of treatement were not affected by simvastatin. Plasma HDL-C levels, however in mice fed 5.0 mg/kg of simvastatin were significantly higher (19.4% upregulation) than that in model group. In addition, administration of simvastatin significantly increased plasma levels of apoA-I (Table 2) and LCAT activities (Figure 2).

**Table 2:** Effect of Simvastatin on plasma lipoprotein lipids and apoA-I concentrations in apoE-/- mice

	Model (apoE-/-)	**Simvastatin **(5 mg/kg)
**Animal number (n)**	**11**	**10**
**TC(mmol/L)**	**24.69 ± 5.34**	**22.07 ± 4.12**
**TG(mmol/L)**	**1.13 ± 0.37**	**1.24 ± 0.47**
**Non-HDL-C (mmol/L)**	**22.06 ± 4.87**	**18.93 ± 3.89**
**HDL-C (mmol/L)**	**2.63 ± 0.35**	3.14 ± 0.34**
**apo-AI(mg/dl)**	**4.24 ± 1.01**	11.58 ± 2.03**

Values are the means ± SD. *compared with model, p < 0.05. **compared with model, p < 0.01

### Effect of simvastatin on the expression of hepatic genes involved in cholesterol metabolism

**Figure 3:**
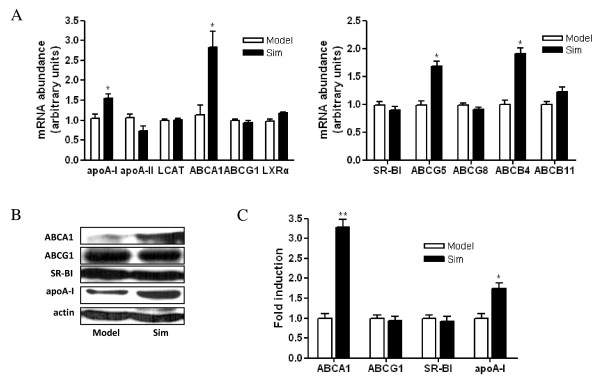
**Effect of simvastatin on the expression of hepatic genes which play roles in cholesterol transport**. A, Effect of simvastatin on the mRNA expression of hepatic genes by real-time PCR. Expression levels of mRNA are indicated as fold differences compared with model mice. B, Effect of simvastatin on the protein expression of hepatic genes by western blots. C, Densitometric quantitation of western blot data (n = 5) by Quantity One software. * P < 0.05, ** P < 0.01 versus model group.

As shown in Figure 3 A, simvastatin treatment increased the mRNA levels of hepatic apoA-I and ABCA1 up to approximate 1.55-fold (P < 0.05) and 2.5-fold (P < 0.05), respectively. The mRNA levels of ABCG5 and ABCB4 were also elevated by simvastatin treatment up to approximate 1.7-fold (P < 0.05) and 1.9-fold (P < 0.05), respectively. In addition, the mRNA levels of ABCG1, apoA-II, SR-BI, LCAT, ABCG8, ABCB11 and LXRα were not affected by simvastatin. This difference in hepatic LCAT mRNA levels was not reflected in plasma LCAT activity, suggesting that post-transcriptional regulation plays an important role in LCAT function. Furthermore, as shown in Figure 3 B and C, the protein levels of ABCA1 and apoA-I were elevated and the levels of ABCG1 and SR-BI were not affected by simvastatin treatment.

### Effect of simvastatin on the expression of transporter genes involved in cholesterol efflux in peritoneal macrophages

**Figure 4:**
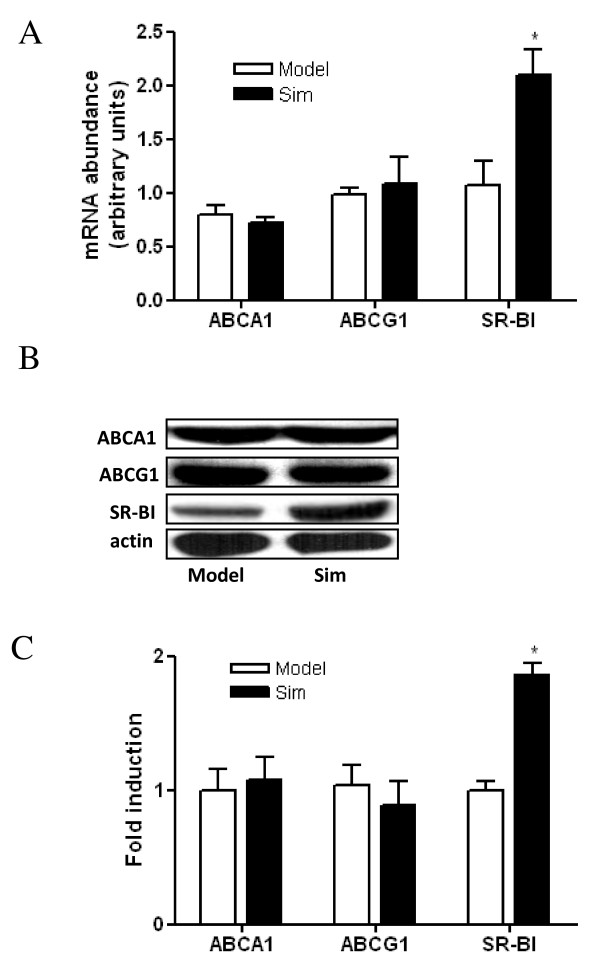
**Effect of simvastatin on the expression of transporter genes involved in cholesterol efflux in peritoneal macrophages**. A, Effect of simvastatin on the mRNA expression of macrophage transporter genes by real-time PCR. Expression levels of mRNA are indicated as fold differences compared with model mice. B, Effect of simvastatin on the protein expression of macrophage transporter genes by western blots. C, Densitometric quantitation of western blot data (n = 5) by Quantity One software. * P < 0.05 versus model group

The effect of simvastatin on the expression of macrophage ABCA1, ABCG1 and SR-BI, which play important roles in cholesterol efflux from peripheral tissues was determined. Real-time PCR and western blot analysis showed that SR-BI expression was significantly increased and the expression of ABCA1 and ABCG1 was not affected by simvastatin treatment (Figure 4).

## Discussion

In the present study, we confirmed simvastatin-treatment not only promoted plasma HDL-C, apoA-I levels, LCAT activities and macrophage SR-BI expression, but also increased the expression of hepatic ABCB4 and ABCG5 besides upregulated hepatic ABCA1 and apoA-I, and reduce atherosclerotic lesion size in apoE-/- mice fed a high-fat diet. It is well-known that statins are first-line pharmacotherapeutic agents for hypercholesterolemia treatment in humans. However the effects of statins on atherosclerosis and lipid levels in mouse models are very paradoxical. Nachtigal P et al. have shown atorvastatin could increase plasma HDL-C and decrease TC levels in apoE/LDL receptor-double-knockout mice fed an atherosclerotic diet [6]. Previous studies [1,7,8] in which the simvastatin administered to the apoE-deficient mice showed that the plasma cholesterol levels of the treated animals fed a chow diet were significantly elevated compared to those of the untreated animals. In our study, we confirmed simvastatin-treatment did not affect plasma TC, TG and non-HDL-C levels, and significantly decreased the atherosclerotic lesion size in apoE-/- mice fed a high-fat diet. Our data was in agreement with Sparrow et al. reported [9] that the administration of simvastatin to apoE-deficient mice fed a high cholesterol diet did not alter their plasma TC levels, Sparrow et al. also found simvastatin decreases aortic cholesterol accumulation in apoE-/- mice besides increased anti-inflammotory effects, but the underlying mechanisms was not yet established. In the present study we found that the simvastatin administrated animals displayed atheroprotection may involved the promoted reverse cholesterol transport by up-regulated HDL-C and related genes expression, including genes important for the formation of HDL-C and genes important for secretion of cholesterol, bile acids, and phospholipid into the bile. In addition, the discrepancy of the effects of statin-treatment on lipid levels in apoE-/- mice might be attributable in part to the possibility that the dyslipidemic profile of apoE-/- mice fed a high-fat diet whose lipid profile is VLDL dominant, is different from and more severe than that fed a chow diet.

Plasma concentrations of HDL-C have strong inverse correlations with risk of atherosclerotic cardiovascular disease, independently of LDL-C levels [10]. ApoA-I is the major protein component of HDL-C in plasma. HDL-C and apoA-I exert anti-inflammatory, anti-oxidant, anti-thrombotic, promoting RCT and vasodilating properties that could all potentially be involved in their anti-atherogenic effects [11]. Here, we showed that simvastatin-treatment increased plasma levels of HDL-C and apoA-I. Our finding was in agreement with another report, which has shown that 8 weeks of simvastatin-treatment has a tendency to increase plasma HDL-C levels in apoE-/- mice fed a high cholesterol diet [12]. Furthermore, apoA-I is mainly synthesized by liver and hepatic ABCA1 exert anti-atherogenic effect via its contribution to HDL-C formation by promoting lipid efflux to apoA-I from hepatocytes [13]. Our previous study have reported that rosuvastatin selectively stimulates apolipoprotein A-I but not apolipoprotein A-II synthesis in Hep G2 cells [14]. And in this study, our results showed that the expressions of hepatic apoA-I and ABCA1 were increased by simvastatin treatment in vivo, these data gave us a clue that the elevated plasma HDL-C level by simvastatin in this study might be closely associated with the enhanced hepatic apoA-I and ABCA1 expression stimulated by simvastatin.

One of the important anti-atherogenic effects of HDL-C is to facilitate the efflux of cholesterol from peripheral tissues and transport it back to the liver in a process called RCT [15]. Recently, several key molecules have been identified to play pivotal roles on RCT [16-18]. Macrophage ABCA1 facilitates cholesterol efflux from cells to lipid-poor apoA-I, but not HDL [16,17], whereas another two macrophage transporters, ABCG1, and SR-BI are involved in the cholesterol efflux from macrophages mediated by HDL, but not apoA-I [18,19]. In our study, we showed that simvastatin induced macrophage SR-BI expression, which might lead to an increased HDL-mediated cholesterol efflux from macrophages. Furthermore, LCAT which bound to HDLs in plasma and play roles in RCT is an enzyme that converts free cholesterol into cholesteryl ester and is critical for the maturation and maintenance of normal HDL metabolism in humans [20] and mice [21]. At present, the molecular mechanism(s) that regulates plasma LCAT concentrations is not well understood. LCAT activity depends in part on the amount of the enzyme in plasma, and in part on the substrate and cofactors available to the enzyme [22], such as apoA-I, an activator of LCAT, and apoA-II, an inhibitor of LCAT activity. Our results showed that simvastatin-treatment did not influence the mRNA expression of hepatic LCAT and apoA-II, but significantly increased plasma LCAT activities, hepatic apoA-I and plasma apoA-I concentrations. The discrepancy in hepatic LCAT expression and plasma LCAT activity might attributable to a post-transcriptional regulation of LCAT or the upregulation of apoA-I, the activator of LCAT, by simvastatin.

Besides macrophage transporters and plasma LCAT, several hepatic transporters are also involved in the process of RCT. The direct selective uptake of HDL-C by the liver is mediated by SR-BI. However, in our study, the expression of hepatic SR-BI was not altered in simvastatin-treated mice. After uptaking of HDL-associated cholesterol by SR-BI, secretion of cholesterol, bile acids, and phospholipid into the bile is regulated by the respective transporters ABCG5, ABCG8, ABCB4 and ABCB11. Here, we showed for the first time that the mRNA expression of ABCG5 and ABCB4 was upregulated by simvastatin-treatment. These finding suggest that simvastatin may exert anti-atherosclerotic effects by stimulating the expression of transporters involved in the process of RCT, including macrophage SR-BI, hepatic ABCG5 and ABCB4. The mechanisms in which simvastatin stimulated higher expressions of RCT related genes in apoE-/- mice deserve further investigation.

## Conclusions

In conclusion, we confirmed here for the first time simvastatin increased the expression of hepatic ABCB4 and ABCG5 besides upregulated ABCA1 and apoA-I in apoE-/- mice. Our data show that in vivo administration of simvastatin effectively attenuates atherosclerotic lesion formation in apoE-/- mice fed a high-fat diet by targeting several pathways involved in atherogenesis. Elevated plasma apoA-I and HDL-C levels, increased plasma LCAT activities, and the stimulation of reverse cholesterol transporter gene expression may all contribute to the beneficial effect of simvastatin. Our findings provide a novel insight into the anti-atherosclerotic effects of simvastatin besides its lipid-lowering effect.

## Competing interests

The authors declare that they have no competing interests.

## Authors' contributions

GS carried out the study design, data collection and analysis, the animal experiment and drafted the manuscript. JL carried out the animal studies and performed real-time PCRs. ZZ helped to carry out animal studies. YY carried out the biochemical analysis. HT performed the atherosclerotic lesion analysis. SY and GL participated in the animal studies. SQ was responsible for the study design, the funding, the data analysis, and the manuscript draft. All authors read and approved the final manuscript.
